# Phytostabilization—Management Strategy for Stabilizing Trace Elements in Contaminated Soils

**DOI:** 10.3390/ijerph14090958

**Published:** 2017-08-25

**Authors:** Maja Radziemska, Magdalena D. Vaverková, Anna Baryła

**Affiliations:** 1 Department of Environmental Improvement, Faculty of Civil and Environmental Engineering, Warsaw University of Life Sciences, Nowoursynowska 159, 02-776 Warsaw, Poland; anna_baryla@sggw.pl; 2 Department of Applied and Landscape Ecology, Faculty of AgriSciences, Mendel University in Brno, Zemědělská 1, 613 00 Brno, Czech Republic; magda.vaverkova@uake.cz

**Keywords:** aided phytostabilization, metal contaminated soil, clay minerals, red fescue, risk minimization

## Abstract

Contamination of soil by copper (Cu) has become a serious problem throughout the world, causing the reduction of agricultural yield and harmful effects on human health by entering the food chain. A glasshouse pot experiment was designed to evaluate the potential use of halloysite as an immobilizing agent in the aided phytostabilization of Cu-contaminated soil, using *Festuca rubra* L. The content of Cu in plants, i.e., total and extracted by 0.01 M CaCl_2_, was determined using the method of spectrophotometry. Cu content in the tested parts of *F. rubra* differed significantly when halloysite was applied to the soil, as well as with increasing concentrations of Cu. The addition of halloysite significantly increased plant biomass. Cu accumulated in the roots, thereby reducing its toxicity to the aerial parts of the plant. The obtained values of bioconcentration and translocation factors observed for halloysite treatment indicate the effectiveness of using *F. rubra* in phytostabilization techniques.

## 1. Introduction

Human health and quality of life are strictly connected with the quality of the natural environment [1]. That is why we can more frequently observe the far-reaching health advantages resulting from the concern for a high quality of the natural environment. The increasing contamination of soils with heavy metals over recent years is one of the most troublesome problems and challenges faced by environmental protection groups [2]. According to the United States Environmental Action Group, this environmental problem threatens the health of more than 10 million people across many countries [3].

Heavy metals comprise contamination that is particularly dangerous due to the specific properties of these elements. They are accumulated in living organisms in an uncontrolled manner and introduced into the food chain, the final link of which are humans [4,5]. One ought to be aware of the fact that the contents of heavy metals in soil that are in line with norms specified in legal regulations do not necessarily provide a full guarantee of ecological safety, especially when contaminated soils are characterized by poor sorption abilities and an acidic pH.

Among the elements most frequently leading to contamination in terrestrial surface ecosystems is copper (Cu) [6,7]. The greatest source of environmental pollution caused by this metal is mining and smelting, as well as the electronic industry [8,9]. In literature, attention has been drawn to large urban centers where the emission of exhaust fumes is a further reason behind the increase in the level of this element [10,11]. An increase in the content of copper in soils is also observed as a result of agriculture, mainly the use of copper-based substances and applying municipal sewage sludge for the fertilization of soils [12]. In the case of an uncontrolled increase in the copper content of soil, metabolic disorders can be observed in plants, the effects of which are stunted growth and development. The toxic effects may be due to limiting the process of photosynthesis by inhibiting the transport of electrons and the synthesis of photosynthetic pigments (mainly chlorophyll a and b), an increase in H_2_O_2_, free radicals OH- and O_2_-, which damage DNA, and disturb the permeability of cell membranes, which consequently increases the emission of potassium and phosphate ions [13]. Cu threatens human health by contaminating soil and groundwater, as well as bodies of water.

**Figure 1:**
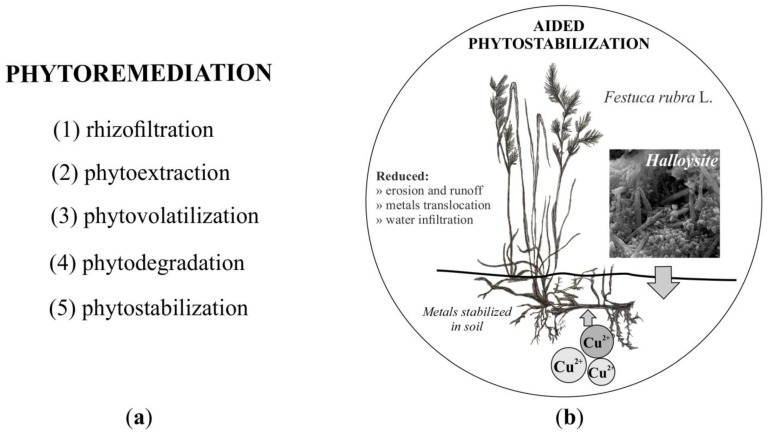
(**a**) Classification of phytoremediation methods; (**b**) concept of aided phytostabilization using halloysite and red fescue (*Festuca rubra* L.).

Phytoremediation can be classified into different applications, such as (1) rhizofiltration; (2) phytoextraction; (3) phytovolatilization; (4) phytodegradation; and (5) phytostabilization (Figure 1a). Aided phytostabilization is a biological method included in the Gentle Remediation Options (GRO), which, among others, are safer and least interfere with the natural environment [14]. This technique is based on the chemical stabilization of heavy metals using various non-organic and/or organic soil additives in connection with adequately chosen plant species [15]. Species which will be resistant to specific conditions present in the soil, such as low pH and high concentrations of heavy metals, ought to be selected. Moreover, they should not accumulate heavy metals in their above-ground parts, thus preventing their further passage to subsequent elements of the food chain, and should be characterized by a fast increase in biomass, ensuring good coverage of the area in a short period of time [16]. An example of such plants are grasses from the fescue family of grasses, which are commonly used to create a vegetation cover in post-mining areas and slag heaps. Various species of grass, such as red fescue (*Festuca rubra* L.) are the most useful in the process of the aided phytostabilization of heavy metals in soils [17]. Some literature reports [18,19] show that *F. rubra* is a suitable species for the revegetation of metal-contaminated soils contaminated by industrial activities such as mining, energy, and fuel production. Furthermore, *F*. *rubra* has the ability to accumulate Cu, Pb, Mn, and Zn from contaminated soils [20,21,22]. The aim of this technique, besides limiting the bioavailability of heavy metals, is also to restore adequate soil quality [23]. Various non-organic materials, such as: CaO, apatite, chalcedonite, septolite, diatomite, dolomite, bentonite, halloysite, hematite or FeO [24,25,26] and/or organic compounds, e.g., brown coal and wood coal, compost, peat, fly ash, woodchips or wood bark [27,28] are used individually or in combination as soil additives.

The search for new sorption materials which can be used as soil additives supporting the soil contaminant immobilizing processes is of key importance in the intensification of heavy metal removal or immobilization processes in soil and the improvement of its quality. The availability, prevalence, price and effectiveness of removing contaminants from the area with the applied sorption material are undoubtedly of significance; hence, the reason behind the new and intensive search for original and effective additives that may be used in processes of aided phytostabilization.

Halloysite [Al_2_Si_2_O_5_(OH)_4_·(H_2_O)] is an aluminum silicate of volcanic origin, characterized by high porosity (60–70%) and specific surface (65–85 m^2^·g^−1^), high ion-exchange capacity thanks to which it has a high ability to absorb heavy metals, and the ease of both chemical and mechanical treatment. Halloysite from Polish deposits is characterized by a specific platy and tubular structure, with a prevalence of the platy fraction. The inside diameter of tubes is 15 nm, while their length can reach up to 1000 nm [29]. Poland is home to one of the largest deposits in the world—the “Dunino” deposit near Legnica (SW Poland), is one of three places in the world, in addition to New Zealand and the USA, with resources estimated at over 10 million tons [30]. It contains mainly halloysite nanotubes (HNT) and nanoplates (HNP) [31].

A solution may be to use new immobilizing soil amendments, i.e., halloysite and red fescue (*Festuca rubra* L.), as proposed by the authors, as plants phytostabilizing the contamination of soil by copper compounds (Figure 1b).

Studies under the conditions of a pot experiment in which variables are reduced by constructing a system corresponding to that found in nature, may in many aspects, support the understanding of phenomena which can occur under natural conditions. Such experiments may serve to optimize phytotechnology prior to its practical application. Based on the above discussion, studies which aimed to determine the possibilities of using raw halloysite in processes of the aided phytostabilization of soil contaminated with copper using *Festuca rubra* L. were undertaken. In order to determine the possibilities for the development of adequate vegetation cover on the contaminated soil, the biomass of the test plant was determined; moreover, the contents of copper in individual organs of *F. rubra* (aerial part and roots) were subject to study, and the degree of copper immobilization in soil was determined by assessing its total concentration and the fraction extractable in CaCl_2_.

## 2. Materials and Methods

### 2.1. Experimental Materials and Methods

The experiments were conducted in 5.0 kg polyethylene pots using soil collected at a depth comprising approximately the top layer (0–30 cm), from a non-contaminated site in an agricultural area of Poland. The soil was characterized by the following physicochemical properties: grain-size distribution: fractions 2.0–0.05 mm 86.6 (%); fractions 0.05–0.002 mm 11.2 (%), fractions 0.002 mm 2.2 (%); pH 5.81; electrical conductivity (µS·cm^−1^) 87.21; hydrolytic acidity (mmol·kg^−1^) 31.21; sum of exchangeable bases, Ca^2+^, Mg^2+^, K^+^, Na^+^ (mmol·kg^−1^) 61.10; cation exchange capacity (mmol·kg^−1^) 94.20; base saturation (%) 65.20; total nitrogen (g·kg^−1^) 0.98; organic carbon (g.kg^−1^) 6.42; ammonia (mg·kg^−1^) 20.32; nitrate (V) (mg·kg^−1^) 2.01; extractable phosphorous (mg·kg^−1^) 43.20; extractable potassium (mg·kg^−1^) 8.72; extractable magnesium (mg·kg^−1^) 31.2; and Cu (mg·kg^−1^) 8.20. Soil was artificially polluted with aqueous solutions of copper (in the form CuSO_4_·5H_2_O, Sigma–Aldrich, St. Louis, MO, USA) and fertilized with a macro- and micronutrient fertilizer mixture (g·kg^−1^) containing K_2_O–26%, P_2_O_5_–12%, N–26%, Mn–0.25%, B–0.013%, Cu–0.025%, Fe–0.05%, and Mo–0.20%. Red fescue (*Festuca rubra* L.) cv. dark was chosen as the test plant. Red fescue seeds, each weighing 5 g, were sown in the pots, germinating five days later. The plants were watered every other day with deionized water to 60% of the maximum water holding capacity of the soil. At the end of the experiment (approx. 42 days after seed sowing), plants were harvested, weighed and separated into above-ground parts and roots. 

Simulated soil contamination with copper introduced in the form of chemically pure aqueous solutions of copper (II) chloride dehydrate (CuCl_2_·2H_2_O, produced at the POCh, Gliwice, Poland) was introduced in the following doses (mg·kg^−1^ of soil): 0 (control), 150, 300, and 600. The Cu salts were dissolved in distilled water and added to the soil. Halloysite was mixed in with the soil in the amount of 3.0% (each). Soils without copper and halloysite (0.0%) were designated as the control. The choice of the amount of halloysite was based on the results of multi-year studies carried out by the authors [15,32,33,34]. The amounts were chosen optimally, so as to maximize the immobilization effect with the lowest possible use of sorbents. The soil samples were thoroughly mixed and pots were placed in a dark room for over two weeks with 70% of water holding capacity to equilibrate the soil mixture. Each treatment was replicated thrice (∑ = 24 pots). Copper was selected as the target contaminant as it is the most common contaminant found in subsurface soils. The content of Cu in Polish soils ranges from 0.2 to 725.0 mg Cu·kg^−1^ d.m. and contamination rates detected near metal smelters in other parts of the world range from 510 to 9700 mg Cu·kg^−1^ [35,36].

### 2.2. Halloysite Characterization

The raw halloysite used in the study was obtained from the “Dunino” strip mine (Intermark Company, Legnica, Poland), and was characterized by a large specific surface area of 49.52 m^2^·g^−1^ [25]. The sample contained ~60% halloysite and ~40% kaolinite as estimated by the formamide test [37]. According to Szczepanik et al. [38], halloysite from this deposit is characterized by the following oxide composition (%) SiO_2_–39.6, Al_2_O_3_–37.0, Fe_2_O_3_–16.1, TiO_2_–2.30, CaO–0.66, MgO–0.13, Na_2_O–0.04, K_2_O–0.05, P_2_O_5_–0.52. The granulometric composition of halloysite is 31% sand fraction (0.05 < d < 2.0), 40% fine particle fraction (0.002 < d < 0.05), and 29% silt fraction (<0.002). Grain sizes over 0.5 mm were not observed. The dominant fraction is that of 0.002–0.05 mm. Bulk density amounted to 0.81 g·cm^−3^. This parameter is significant when it comes to storing and transporting material. The higher the value of the bulk density, the less free spaces between the particles of the sorbent of the same apparent density. Bulk density is dependent on the distribution of the size and shape of the particles, as well as their relative density which has an indirect connection with the type of sorbent and its porosity. The moisture content amounted to 2.5%. Moisture content is a parameter that is dependent on the structure of the material, which influences its thermal conductivity, which increases significantly along with an increase in moisture content.

### 2.3. Soil Sampling and Analyses

Upon completing the experiment, each sample was air dried and ground to pass through a sieve of 2 mm mesh size, and prepared for analysis in such a way, kept in clean, appropriately marked polyethylene containers. Before setting up the experiment, the following soil properties were determined: pH and electrical conductivity (EC) of soil samples were measured in an aqueous suspension of 1:5 (w/v) using a pH meter (Model pH/LF 12, Schott, Germany) and conductivity meter (Model pH/LF 12, Schott, Germany); total exchangeable bases (TEB) Na^+^, K^+^, Mg^2+^, and Ca^2+^ were determined by standard procedures given by Klute [36] and their contents were determined using an Atomic Absorption Spectrophotometer (iCE-3000, Thermo Scientific, Waltham, MA, USA); hydrolytic acidity (HAC) by Kappen’s method, with soil samples treated with 0.5 M·dm^−3^ Ca-acetate solution adjusted to a pH of 8.2 in the ratio of 1:2.5 [39]; cation exchange capacity (CEC) from the formula: CEC=HAC+TEB, and percentage base saturation (V)—from the formula: BS = 100·TEB/CEC^−1^; phosphorous and potassium content from the Egner–Riehm method [40]; magnesium content—atomic absorption spectrometry method following extraction using the Schachtschabel method [41]; nitrogen content in soil—by Kjeldahl’s method [42]; ammonium content of the soil—with an ammonia electrode as ammonium formed by Kjeldahl digestion [39]; organic matter (OM)—according to Tiurin’s method [43]; copper —with an iCE-3000 spectrophotometer (Thermo Scientific, Waltham, MA, USA) using U.S. EPA method 3051. Certified reference material (Sigma Aldrich Chemie GmbH, No. SQC001, St. Louis, Missouri, MO, USA) was used for analyses. The exchangeable soil metal fractions of copper were determined using 0.01 M CaCl_2_ (1:10 soil to extractant ratio) after agitation for 2 h at 20 °C. The extract was separated from the solid residue by centrifugation for 15 min [44]. Triplicates were performed for each sample.

### 2.4. Plant Material Analyses

Plants were pulled out carefully from the pot to avoid any damage to the roots. Harvested *F. rubra* samples were cleaned with ultra-pure water, air-dried at room temperature for two weeks, and then ground up to a fine powder using an analytical mill (Retsch type ZM300, Hann, Germany) and kept at ambient temperature, protected from light in clean containers for subsequent chemical analysis. The roots and shoots were oven-dried at 55 °C to a stable weight, with the dry biomass recorded. A representative subsample was mineralized in nitric acid (65% w/w, Chempur, Poland) and hydrogen peroxide (30% w/w, Merck, Darmstadt, Germany) using a microwave oven (Milestone Start D, Milan, Italy). After filtration, the products of digestion were adjusted to 100 mL volume with deionized water. Extracts were analyzed for total Cu concentrations determined by the Atomic Absorption Spectrometry (AAS) method using an iCE-3000 spectrophotometer (Thermo Scientific, Waltham, MA, USA). Five-point calibration was performed with standard solutions. Each sample was processed in triplicates. Laboratory equipment was acid-washed (10% HNO_3_) and rinsed with deionized water. 

### 2.5. Phytotoxicity Study

The aim of the experiment carried out using the germination test and early plant growth test PhytotoxkitTM (Microbiotests, Nazareth, Belgium) was to determine whether the addition of halloysite to soil polluted with Cu will have an influence on the development of the test plants. This test is based on measuring the germination and early growth ability of white mustard roots (*Sinapis alba* L.) in the analyzed sample in comparison to the values obtained for a reference soil containing 85% sand, 10% kaolin, 5% peat, at pH 6.5–7.0 controlled with CaCO_3_. The test plates were placed vertically in a holder incubated at 25 °C for 3 days. At the end of incubation, a digital picture was captured for each of these test plates with the germinated plants. The assessment covered 3%, 9%, 30% and 100% concentrations of halloysite. Measurements were taken in three repetitions for each amount of sorbent. The measurement of the length of the root for the Phytotoxkit test was carried out using Image Tool 3.0 software for Windows (UTHSCSA, San Antonio, TX, USA). The percentage of root growth inhibition (RI) were calculated with Formula (1) [45]:

(1) $$\mathrm{RI}=\frac{a-b}{a}100,$$

where a is the mean seed germination or root length in the control soil and b is the mean seed germination and root length in the test [46].

### 2.6. Properties of Halloysite

The assessment of the physical properties of material, such as the grain size, bulk density and moisture content, was carried out. Studies of the mechanical composition were carried out with the sieve method when wet, as well as the sedimentation method. Bulk density of free-flowing sorbent was described as the ratio of the mass of loosely poured material to the volume taken up, expressed in a unit of mass per unit of volume. The value of bulk density was indicated with a method in accordance with PN-80/C-04532 method B [47].

Bulk density of freely poured product (X) was calculated in g·cm^−3^, according to Formula (2):

(2) $$X=\frac{\mathrm{mass}\;\mathrm{of}\;\mathrm{halloysite}}{\mathrm{capacity}\;\mathrm{of}\;\mathrm{measurement}\;\mathrm{cylinder}}$$

The moisture content of the material was assessed using the dry oven test (temperature 105 °C). The obtained results were calculated using Formula (3), where: Mw, is the mass of the sample prior to drying (g), Ms, is the mass of the sample after drying (g):

(3) $$w=\frac{\mathrm{Mw}-\mathrm{Ms}}{\mathrm{Ms}}100$$

### 2.7. Calculation and Statistical Analysis

Two indices, translocation factor (TF) and bioconcentration factor (BCF), used for evaluating the phytostabilization potential of a plant, were calculated using the formulas given in Equations (4) and (5) [15].

(4) $$\mathrm{BCF}=\frac{\mathrm{Metal}\;\mathrm{concentration}\;\mathrm{in}\;\mathrm{roots}}{\mathrm{Metal}\;\mathrm{concentration}\;\mathrm{in}\;\mathrm{soil}}$$

(5) $$\mathrm{TF}=\frac{\mathrm{Metal}\;\mathrm{concentration}\;i\;\mathrm{above}-\mathrm{ground}\;\mathrm{parts}}{\mathrm{Metal}\;\mathrm{concentration}\;\mathrm{in}\;\mathrm{roots}}$$

Experiments were performed in triplicates and the values presented as the means ± standard deviation. The data were analyzed using Statistica software (version 10.0, San Diego, CA, USA). Significant differences (*p* < 0.05) between the mean values of different treatments were compared and evaluated using Duncan’s multiple range test.

## 3. Results

### 3.1. Effect of Copper Contamination and Halloysite Application on Biomass of F. rubra

**Figure 2:**
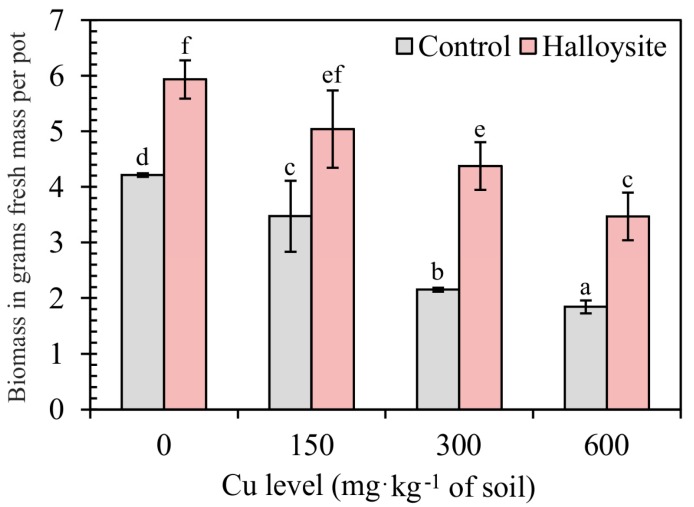
Effect of copper (Cu) and halloysite on the above-ground biomass of *F. rubra* in grams of fresh mass per pot. Error bars are ± standard error (n = 3). Bars marked with different letters differ significantly for the same Cu exposure (*p* < 0.05) according to the Duncan test.

The effects of halloysite on the biomass production of *F. rubra* grown in a Cu-contaminated soils are shown in Figure 2. In this study, *F. rubra* germinated and grew successfully. Moreover, the plants did not show any visible symptoms of copper toxicity or nutrient deficiency when grown in the soil contaminated with halloysite. Above-ground parts of *F. rubra* in the control objects (without the addition of halloysite) were characterized by high sensitivity to soil contamination with Cu, as confirmed by the existence of a negative correlation between crop yield and increasing soil contamination with copper compounds. When the concentration of Cu in soil remained at 600 mg·kg^−1^, it significantly decreased the biomass of plants, i.e., by 44%. Halloysite positively influenced the growth and yield of the above-ground parts of *F. rubra* as compared to the control objects. It was most beneficial to the increase (+ 62%) in the amount of biomass in the test plant at a Cu dose of 300 mg·kg^−1^ of soil.

### 3.2. Metal Accumulation and Translocation Efficiency in F. rubra

**Figure 3:**
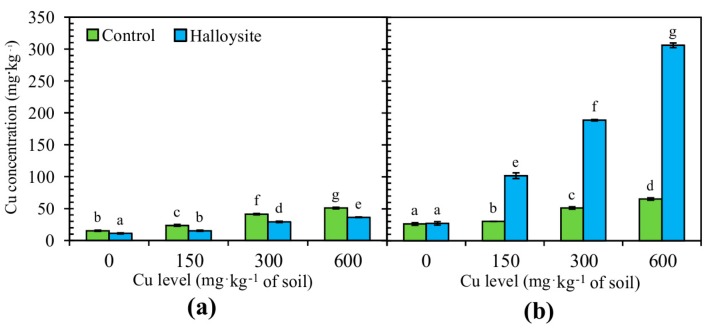
Copper concentration (mg·kg^−1^, dry weight basis) in the above-ground part (**a**) and roots (**b**) of *F. rubra* at the end of the trial. Error bars are ± standard error (n = 3). Bars marked with different letters differ significantly for the same Cu exposure (*p* < 0.05) according to the Duncan test.

The influence of different treatments on copper accumulation and distribution in the *F. rubra* organs is reported in Figure 3. The accumulation of Cu content was much higher in the roots than in the above-ground parts, and this major advantage indicates the potential of *F. rubra* as a phytostabilizer. In the control (without halloysite), Cu concentrations (in mg·kg^−1^ dry weight) varied between 12.23 and 50.88 in the above-ground parts and between 26.15 and 65.38 in the roots. The present research reveals that the application of halloysite influenced the average Cu content in *F. rubra*. The highest reduction of Cu contents (35%) was observed in the above-ground parts of the tested plant species grown in soil contaminated with 150 mg·kg^−1^ Cu, as compared to the uncontaminated soil. The roots of the test plant contained 5 times more Cu as compared to the control group when soil was contaminated with 600 mg·kg^−1^ Cu.

**Figure 4:**
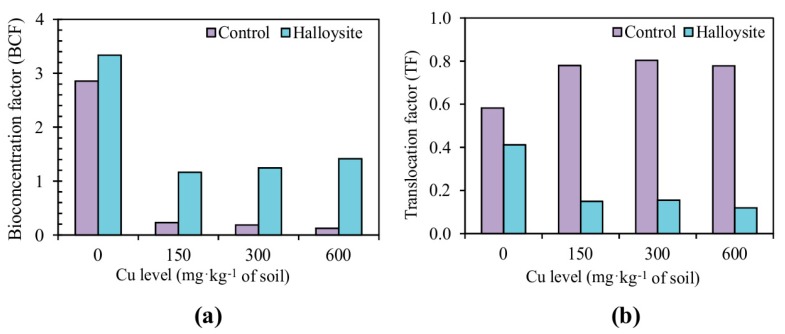
Effects of applying halloysite on copper accumulation (BCF) (**a**) and translocation (TF) (**b**) in *F. rubra* (grown in Cu-contaminated soil).

In order to evaluate the metal accumulation efficiency in *F. rubra*, the bioaccumulation factor (BCF) and the translocation factor (TF) were calculated. The BCF values (the Cu ratio between roots and soil) for *F. rubra* are given in Figure 4a. BCF values of *F. rubra* ranged from 0.12 to 0.23 in the control series without halloysite application, whereas upon adding it to soil contaminated with Cu, the values of BCF were from 1.16 to 3.34. TF has been defined as the concentration of Cu in the aerial parts of a plant divided by the concentration of Cu in plant roots. The ratio can be used to evaluate the translocation effects within *F. rubra*. The comparisons of the TF for the different treatments are shown in Figure 4b. *F. rubra* TF in the control series varied from 0.58 to 0.80, ranging from 0.12 to 0.41 in the series with halloysite application to soil.

### 3.3. Phytotoxicity Evaluation of Halloysite

**Figure 5:**
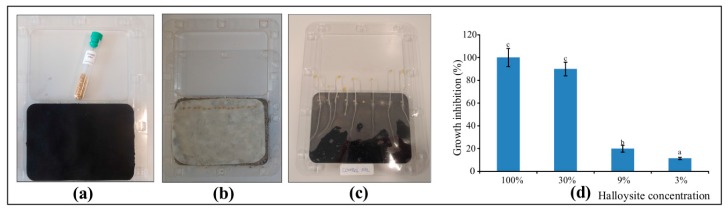
Experiment setup: *Sinapsis alba* L. seeds (**a**), germination (**b**), growth after three days (**c**), the growth inhibition results (**d**). Error bars represent ± standard error (n = 3). Bars marked with different letters differ significantly (*p* < 0.05) according to Duncan’s test.

Figure 5 presents the experiment setup and the growth inhibition results. The completed studies showed an unfavorable interaction between the applied doses of halloysite and the length of roots of the tested plant at a halloysite concentration of 100%. Impeded growth of the root of *Sinapis alba* L. at a concentration of 9% added halloysite was also significant, and found to be at 19.82%. A 3% concentration of halloysite had the least inhibiting effect on the elongation of roots of the test plant (inhibition value was 11.34%). At this concentration, changes in the analyzed plants were not noted (i.e., deformations, discolorations, significantly smaller or greater heights of seedlings as compared to the control group).

### 3.4. Effects of Halloysite on Soil Chemical Properties

**Figure 6:**
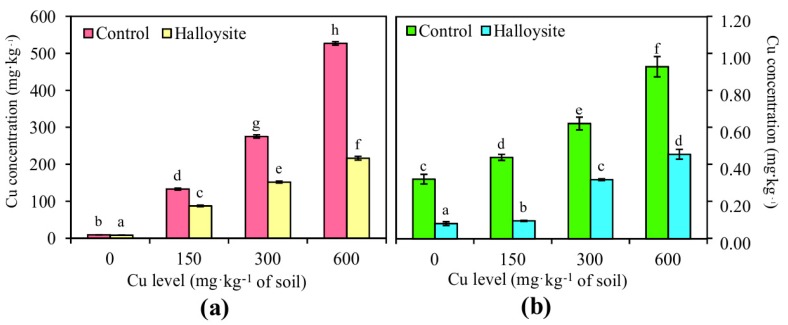
Total (**a**) and CaCl_2_-extractable (**b**) Cu fractions in soil with the different soil treatments (mean ± SD, n = 3). Values in columns marked with the same letter do not differ significantly (Duncan’s test, *p* > 0.05).

**Figure 7:**
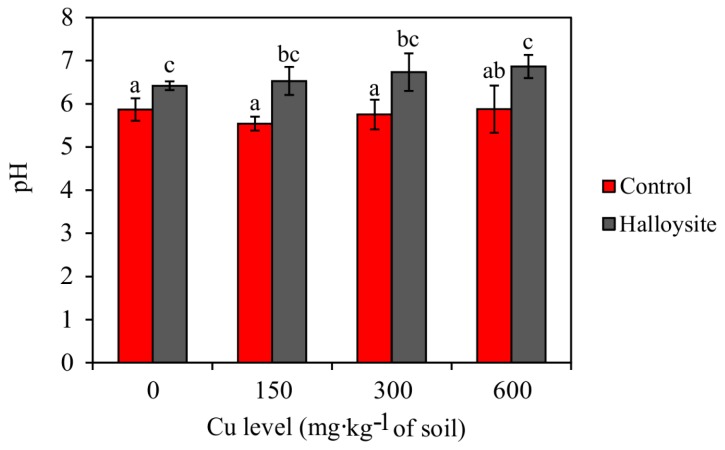
Results of soil pH obtained for the tested amendment; (mean ± SD, n = 3). Values in columns marked with the same letter do not differ significantly (Duncan’s test, *p* > 0.05).

Total and CaCl_2_-extractable Cu concentrations have been presented in Figure 6. The content of available Cu in treatments with halloysite was significantly lower than the total content. The application of halloysite led to a significant decrease in total Cu concentrations in soil as compared to the control pots. At the highest dose of Cu (600 mg·kg^−1^ soil), an over twofold decrease in the total content of Cu was observed in soil subjected to analysis. This suggests that soils treated with the application of halloysite exhibit a greater ability to desorb Cu from the soil in comparison to soil without the amendment. In the presented research, the pH of soil solutions increased following the addition of halloysite (control: 5.76 ± 0.12; halloysite: 6.64 ± 0.06) (Figure 7). In the series lacking halloysite, increasing doses of Cu contamination caused a successive increase in the pH of soil.

## 4. Discussion

Plants react to soil contamination with heavy metals very quickly since, as a result of changes in the chemical composition of soil, the selective accumulation of heavy metals in their various parts takes place [48]. Changes taking place in the plant, which include: decreased biomass, root length and shoot length, are common indicators of the toxic effects of heavy metals [49], while changes at the level of cells and tissues are usually the result of the direct effects between the metal and metabolism [50]. Plants during their growth influence the concentration of heavy metals in contaminated soils in two ways: (1) by blocking the absorption of heavy metals to plant tissues by the roots; (2) by the ability to take up, store and immobilize heavy metals by binding them with biologically active particles. Benimeli et al. [51] investigated the accumulation of Cu^2+^ by roots, shoots and leaves of *Zea mays* in various concentrations of Cu and found that the growth of tested plants was not stunted by exposure to 100 mg·kg^−1^ Cu^2+^. Other authors using additives (i.e., zeolite, calcium oxide, bentonite, chalcedonite, diatomite, dolomite) to immobilize heavy metals in soil confirm their positive effect on the crop yield of tested plants [52,53,54,55]. In the present experiment, halloysite applied to soil contaminated with Cu significantly increased test plant yield. 

The accumulation of heavy metals in the above-ground as well as below-ground parts of plants increases along with an increase in the concentration of the metal coming from the outside environment [56,57]. Comparing the results of the present studies with the reports of other authors [58,59], Cu, as a potential element characteristic of soils contaminated with heavy metals, has already been the subject of studies using the phytostabilization technique. However, using mineral amendments in the process of aided phytostabilization, as well as in connection with the occurrence of Cu in selected chemical forms (total or potentially bioavailable) in soil, as well as its accumulation by various plant species has not been well understood up until now. Our results are in accordance with the previous findings of Radziemska et al. [15,25]. We found that above-ground parts of *F. rubra* in pots with the addition of halloysite were characterized by lesser sensitivity to soil contamination with copper. Similar results were observed by Sinnett et al. [60] and Chapman et al. [61], who pointed out that red fescue appears to have a high tolerance for heavy metal contamination. The plant roots have the ability to modify the area of the rhizosphere, increasing or limiting the uptake of elements. Under conditions of a deficiency of nutrients, they increase the bioavailability of elements, whereas in the case of toxic contents of bioavailable metals, they lead to their immobilization in the soil. This occurs because metal uptake by the roots is as rapid compared with its transport to other plant tissues. The analysis of results presenting Cu contents in individual parts of the test plant confirms the thesis commonly presented in the literature that the highest accumulation of the majority of metals, including copper, takes place in the roots followed by the aerial parts [35]. Shorrocks and Alloway [62] found that Cu toxicity in crops occurred when the concentration in the shoot was more than 20 mg·kg^−1^ in the dry matter. In this study, as in the studies of other authors [15,25,32,63], the application of reactive materials (i.e., diatomite, chalcedonite, zeolite, dolomite, limestone, halloysite, zeolite, bentonite) led to a significant decrease in the concentrations of Cu and other heavy metals in the above-ground parts of the tested plant.

Completing the assessment of phytoaccumulation properties of *F. rubra* are the determined translocation factors (TF) and bioconcentration (BCF) factors. Both parameters depend on the physicochemical properties of the soil matrix and plant species or plant parts. TF quantifies plant defence mechanisms that tend to restrict inorganic contaminants to the roots [63]. The translocation and absorption of essential trace elements occurs actively in plants [64]. In a trial conducted by Sun et al. [54], the addition of bentonite clearly inhibited heavy metal uptake and translocation. The TF values for ryegrass varied minimally, i.e., 0.058–0.065, in an experiment by Abad-Valle et al. [65], in which sepiolite was added to soil contaminated with heavy metals. Also in another experiment by Radziemska et al. [15], mineral additives in the form of diatomite, chalcedonite, dolomite, and limestone exerted a significant influence on the values of TF and BCF. Plants with a BCF greater than one and TF less than one have the potential to be used for phytostabilization [35]. In the presented study, in all analyzed variants of contamination and additions of halloysite, BCF values fell within the range of 1.16–3.34, whereas TF values were between 0.12 and 0.41. Halloysite is an effective way to mobilize trace elements in soil and decrease metal uptake by plants. Sorption, ion exchange, redox processes, the formation of stable complexes with organic ligands and their precipitation can all take place in heavy metal immobilization processes, thus leading to a reduction in the amount of metals directly available to plant roots.

The reason for determining the copper availability for plants was necessary, on one hand, to determine its total content in the soil in order to enable comparison to the earlier works of other authors [66], and on the other, to determine its bioavailable, chemical forms, among which is, e.g., extracted in CaCl_2_. Some elements are by nature, more mobile than others, this being greatly dependent on their soil chemical behavior. Cu ions are among the microelements characterized by the lowest mobility in soil. Released during the aeration process, Cu^2+^ ions bind with soil organic substance, which strongly influences the assimilability of Cu by plants [67]. Increased bioavailability of Cu to plants is caused by increased soil acidity. In soils characterized by a low pH, the mobility of cation forms of copper, i.e.,Cu^2+^, Cu^+^, CuOH^−^ and Cu(OH)_2_^+2^ is greater [35]. Plants can readily uptake the soil-available Cu, although the available Cu accounts for only a small part of the total Cu in the soil. By introducing substances that are capable of increasing the sorption capacity of soils (e.g., halloysite), the amount of absorbable forms of heavy metals in the soil can be decreased. As shown in Figure 6, the available Cu concentration after the addition of halloysite was significantly lower as compared to soil which halloysite had not been added to. This reduction of the CaCl_2_-extractable fraction of heavy metals is one of the main goals of phytostabilization, as it decreases the environmental hazards associated with contamination [15]. Unfortunately, it was not possible to directly compare the results obtained in studies of other authors as, in the majority of cases, in discussions on the accumulation of a given element, including copper, the authors refer to the total content of this element in the soil.

A significant factor determining the solubility of heavy metals in soil is the pH, influencing among others: the assimilation of nutrients by plants and the course of physical–chemical processes in soils. Increasing soil pH by applying alkaline amendments can reduce the solubility and mobility of soil heavy metals because the negative charges of soil colloids increase with increasing pH, resulting in increased sorption of positively charged metal cations [68]. This trend has been found in a variety of studies, including sorption tests, which suggest that the sorption of metallic trace elements increases with increasing soil pH [69]. Decreasing soil pH, on the other hand, leads to lowered permanence of bonds, which results in the disintegration of the sorption complex in soil, as well as the washing out of basic cations to deeper layers of the soil profile. Generally, halloysite increases soil pH and favors the formation of oxides and metal carbonate precipitates, which decrease metal solubility. Pointing to the studies of other authors, such as Ye et al. [70], Jen et al. [71] ad Abad-Valle et al. [66], who applied mineral sorbents to soils contaminated with heavy metals, a significant increase in the pH values of soil can be observed; such a relationship was also noted by the authors of the present study.

## 5. Conclusions

Although heavy metal immobilization treatments in soil do not lead to a complete reduction in their content, they effectively limit ecological risk, and thus, the risk to human health. The level of Cu in the soil matrix, as well as its availability and mobility are crucial to its future in the environment and may determine the success of remedial action, such as in phytostabilization techniques. Carrying out a complex analysis of the soil along with identifying the contents of Cu in individual plant organs growing on contaminated soils should be deemed necessary. Considering that all studies enabling the finding of anomalies based on pilot studies, including those under controlled conditions, are a valuable source of information prior to transferring new solutions to in situ conditions, this has importance for all kinds of discussions regarding the level to which people, plants and animals are being exposed to the dangers of heavy metal contamination in polluted areas.

The present study clearly demonstrates novel evidence with respect to the aided phytostabilization of Cu-contaminated soils, demonstrating that the combined effect of *F. rubra* and halloysite application may be a suitable approach to reducing environmental risk. The biomass of tested plants in particular organs depended on the dose of a Cu contaminant and halloysite incorporated into the soil. In the series without halloysite, Cu had a negative effect on the growth and development of *F. rubra*. In this experiment, Cu accumulated predominantly in the roots of the tested plant. The application of halloysite as a soil amendment tended to reduce the soil mobile fraction of Cu more in comparison to un-amended soil.

Further studies, especially in field conditions, are highly recommended to confirm the efficiency of *F. rubra* and halloysite for the aided phytostabilization of soils contaminated with Cu, because the current study was performed under greenhouse conditions, thus, a controlled environment.
